# Obesity Hypoventilation Syndrome and Postsurgical Outcomes in a Bariatric Surgery Cohort

**DOI:** 10.1007/s11695-022-06073-1

**Published:** 2022-05-11

**Authors:** Janna R. Raphelson, Christopher N. Schmickl, Christine Sonners, Kimberly Kreitinger, Eduardo Grunvald, Santiago Horgan, Atul Malhotra

**Affiliations:** 1 Division of Internal Medicine, UC San Diego Health, San Diego, CA 92109 USA; 2 Division of Pulmonary, Critical Care, & Sleep Medicine, UC San Diego Health, San Diego, CA 92109 USA; 3 Division of General Internal Medicine, UC San Diego Health, San Diego, CA 92109 USA; 4 Bariatric and Metabolic Institute, UC San Diego Health, San Diego, CA 92109 USA; 5 Department of Surgery, UC San Diego Health, San Diego, CA 92109 USA

**Keywords:** Bariatric surgery, Respiratory, Obesity surgery, Weight loss

## Abstract

**Purpose:**

Patients with obesity and elevated serum bicarbonate suggesting obesity hypoventilation syndrome (OHS) undergoing bariatric surgery may represent a unique subgroup. Information regarding surgical outcomes in this population remains limited. We sought to test the hypothesis that an elevated bicarbonate would be an important predictor of perioperative complications (i.e., length of hospital stay) and postsurgical outcomes (i.e., weight loss at 1 year).

**Materials and Methods:**

Consecutive patients undergoing bariatric surgery between January 2015 and December 2018 were included. Patients with a preoperative serum bicarbonate ≥ 27 mEq/L were classified as suspected OHS.

**Results:**

Of 297 patients, the prevalence of suspected OHS based on an elevated bicarbonate was 19.5% (95% CI: 15.3 to 24.6%). Length of hospital stay was similar in the suspected OHS and non-OHS control group (1.50 vs 1.49 days, *P* = 0.98). The achieved weight loss from peak preoperative weight to 1 year post-surgery was less in the suspected OHS vs the control group (4.2% [95% CI 1.6 to 6.8]; *P* = 0.002).

**Conclusion:**

Patients with serum bicarbonate ≥ 27 mEq/L as a surrogate marker for OHS experienced weight loss that was significantly less than their normal serum bicarbonate counterparts, but still achieved weight loss deemed clinically important by current guidelines. We observed no significant difference in length of hospital stay at time of surgery.

**Graphical abstract:**

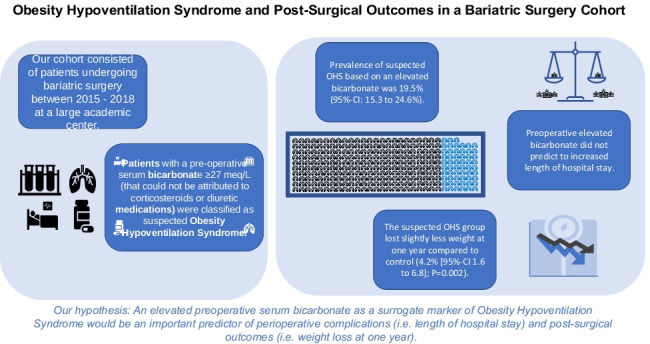

## Introduction

The obesity pandemic has been a major challenge since most conservative therapies have been largely ineffective [1–3]. Increasing emphasis has been placed on bariatric surgery since it has been shown to improve outcomes in those afflicted [4–6]. Such interventions, however, clearly have risks as well as benefits. Considerable focus has been placed on approaches to reducing perioperative risk. In addition, postoperative outcomes can be quite variable with minimal ability to predict which patients are likely to experience benefits or complications.

A subset of patients with obesity suffers from obesity hypoventilation syndrome (OHS) [7], defined by elevated PaCO_2_ in people with obesity without another explanation. Studies have suggested that roughly 30% of patients with a body mass index of 40 kg/m^2^ or greater suffer from OHS, although the entity is likely markedly underdiagnosed. The American Thoracic Society (ATS) Clinical Practice Guideline on the subject recommends weight loss interventions in this population aimed at 25–30% sustained total body weight loss [7], but results of appropriate interventions, such as bariatric surgery, to achieve such weight loss remain understudied [5, 8]. Some experienced clinicians have suggested that weight loss over time may be less in OHS patients following bariatric surgery as compared to matched individuals with similar BMI; however, rigorous data and underlying mechanisms are lacking. Part of the reason for the underdiagnosis of OHS is the lack of arterial blood gas assessments, which are no longer frequently nor routinely performed as part of standard perioperative risk assessment. The ATS guideline suggests using serum bicarbonate levels to identify patients at risk for OHS and select them for further evaluation [7]. The guideline concluded that, in most patients, serum bicarbonate level of < 27 mEq/L effectively rules out hypoventilation with some degree of confidence [7]. Basic metabolic panels are commonly included in most preoperative assessments making serum bicarbonate a practical and available screening tool. Metabolic alkalosis is multifactorial in many cases, however, and such factors as glucocorticoid use and/or diuretic treatments need to be considered before assuming hypoventilation [9–11].

Patients with OHS tend to have a poor prognosis, as shown in multiple epidemiological studies [12–15]. Such patients may be at risk of postoperative respiratory failure due to concerns about extubating patients with hypercapnia [16–18]. In addition, exercise tolerance may be impaired in OHS, perhaps as a function of underlying pulmonary hypertension [19–21]. As a result, diet and exercise may not be as feasible in OHS patients compared to individuals without OHS perhaps effecting postoperative weight loss. Clinical experience by our obesity medicine and bariatric surgery teams indeed suggested that weight loss was attenuated. The Longitudinal Assessment of Bariatric Surgery (LABS) data [16] suggest that obstructive sleep apnea was a risk factor for perioperative complications of bariatric surgery, but the impact of OHS was not evaluated to our knowledge.

Based on this conceptual framework, we sought to test the hypothesis that elevated bicarbonate (as a possible surrogate for obesity hypoventilation syndrome) was a marker of a poor prognosis for patients undergoing bariatric surgery. We examined both in-hospital complications based on length of stay and longer-term outcomes based on net weight loss at 1 year. We used a cohort that we have developed for this purpose and with which we have previously assessed preoperative questionnaires for sleep apnea [22]. Contemporary data are imperative to draw meaningful conclusions since patients undergoing bariatric surgery in the modern era have lower body mass indices and fewer comorbidities than prior years, in part due to emphasis on prevention rather than treatment of obesity-related complications.

## Methods

The study protocol was approved by the institutional review board. Prospectively collected data were analyzed for the analysis in this cohort study. We included consecutive patients who underwent bariatric surgery between January 2015 and December 2018 and had a preoperative serum bicarbonate value available (three patients were excluded as preoperative bicarbonate levels were not available). Patients with a preoperative serum bicarbonate ≥ 27 mEq/L were classified as suspected OHS based on the European Respiratory Society Guidelines. Eight patients with a bicarbonate ≥ 27 mEq/L who were using glucocorticoids or diuretics were excluded from the cohort as these medications may raise serum bicarbonate levels independent of OHS. We manually obtained information including demographics, preoperative serum bicarbonate, diuretic or glucocorticoid use, major comorbidities, and length of hospital stay at time of bariatric surgery. Percent postoperative weight loss at 1 year was calculated from the patient’s maximum preoperative weight in the year preceding surgery as preoperative weight loss is often required. Postoperative weight was obtained from follow-up postoperative clinic visit data ranging from 9 to 15 months after surgery. All procedures performed in studies involving human participants were in accordance with the ethical standards of the institutional and/or national research committee and with the 1964 Helsinki declaration and its later amendments or comparable ethical standards. For this type of study, formal consent is not required.

### Statistical Analysis

For univariable analyses, continuous and categorical variables were compared using *t*-tests and Fisher’s exact tests, respectively. Further, to account for baseline covariates (age, sex, body mass index, hypertension, and ethnicity), we used multivariable linear and logistic regression. To identify predictors with serum bicarbonate ≥ 27 mEq/L and suspected OHS, we used stepwise logistic regression based on the Akaike information criterion (AIC). All analyses were performed in R (3.6.1) using a *P* value of < 0.05 to denote statistical significance.

## Results

**Fig. 1:**
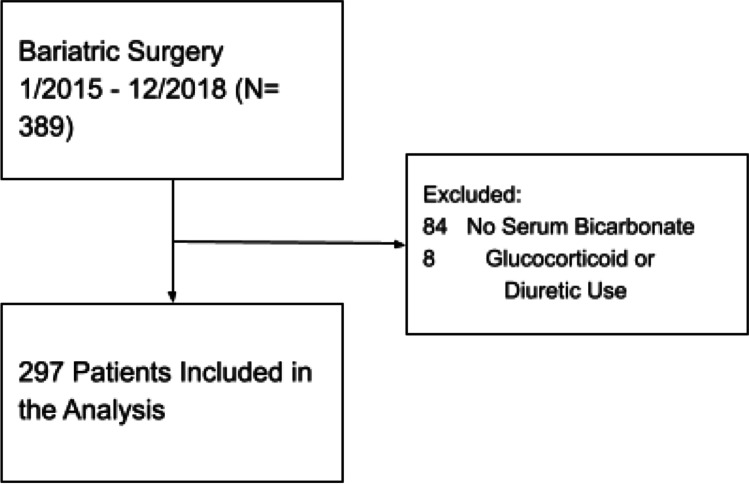
Study flowchart

Of the 297 bariatric surgery patients who were included (Fig. 1), 58 were classified as suspected OHS based on an elevated serum bicarbonate level (without glucocorticoid or diuretic use). The estimated serum bicarbonate ≥ 27 mEq/L and suspected OHS prevalence in this cohort was 19.5% (95% CI: 15 to 25%). Elevated bicarbonate patients were slightly older than normal bicarbonate/non-OHS patients (mean age 44 vs 41 years, *P* = 0.043), but otherwise, both groups had similar characteristics (Table 1). Of the total cohort, 81% identified as female, which is consistent with bariatric surgery utilization rates across the country. The predominant obesity surgery undergone in our study cohort was laparoscopic sleeve gastrectomy. A minority of patients underwent gastric bypass procedure, and prevalence of bypass was proportional in both the elevated and normal bicarbonate groups (2.9 and 3.0%, respectively). In terms of ethnicity, 35% of patients identified as White, 8.4% as Black, 47% as Hispanic, 2.7% as Asian, and 6.7% as other. Comorbid hypertension was present in 53% of patients.

**Table 1:** General characteristics

		Based on bicarb ≥ 27		
Characteristic	Overall, *N* = 297^1^	No OHS, *N* = 239^1^	OHS, *N* = 58^1^	*p* value^2^
Age (*y*)	42 (11)	41 (11)	44 (11)	0.043
Sex				> 0.99
Male	57 (19%)	46 (19%)	11 (19%)	
Female	237 (81%)	190 (81%)	47 (81%)	
Unknown	3	3	0	
Ethnicity				0.87
White	105 (35%)	83 (35%)	22 (38%)	
Black	25 (8.4%)	22 (9.2%)	3 (5.2%)	
Asian	8 (2.7%)	6 (2.5%)	2 (3.4%)	
Hispanic	139 (47%)	112 (47%)	27 (47%)	
Other	20 (6.7%)	16 (6.7%)	4 (6.9%)	
BMI (kg/m^2^)	46 (8)	46 (9)	46 (7)	0.65
Max preop weight	134 (28)	135 (29)	132 (24)	0.57
Unknown	1	1	0	
Hypertension				0.38
No HTN	140 (47%)	116 (49%)	24 (41%)	
HTN	157 (53%)	123 (51%)	34 (59%)	
Bicarbonate	24.76 (2.11)	24.01 (1.54)	27.86 (1.08)	< 0.001
AHI (/h)	20 (25)	19 (24)	26 (29)	0.22
Unknown	120	91	29	
SpO_2_ Nadir (%)	82 (8)	82 (8)	79 (7)	0.044
Unknown	123	93	30	

^1^Mean (SD); *n* (%).^2^Welch two-sample *t*-test; Fisher’s exact test

**Fig. 2:**
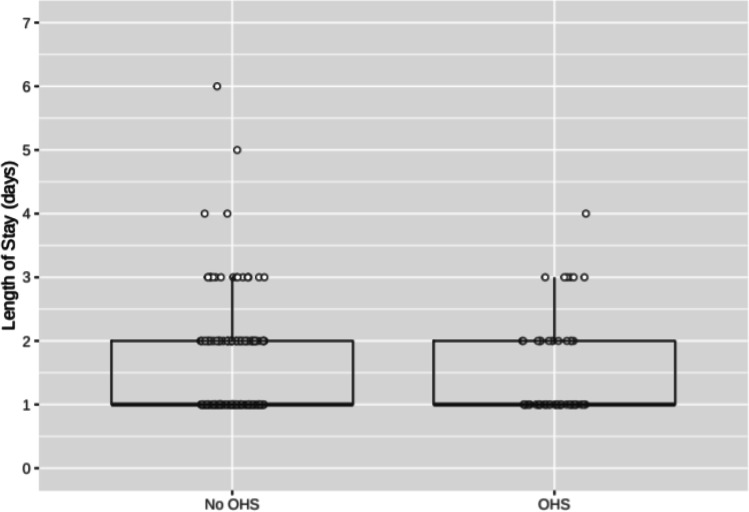
Length of hospital stay in OHS vs non-OHS patients

As shown in Fig. 2, mean length of hospital stay (LOS) was very similar for patients with and without elevated bicarbonate and suspected OHS (1.50 vs 1.49 days, *P* = 0.98). LOS data were non-normally distributed, but results were similar after log-transformation (not shown) or when comparing the fraction of patients whose LOS was greater than 1 day (36% OHS vs 38% non-OHS, *P* = 0.88).

**Fig. 3:**
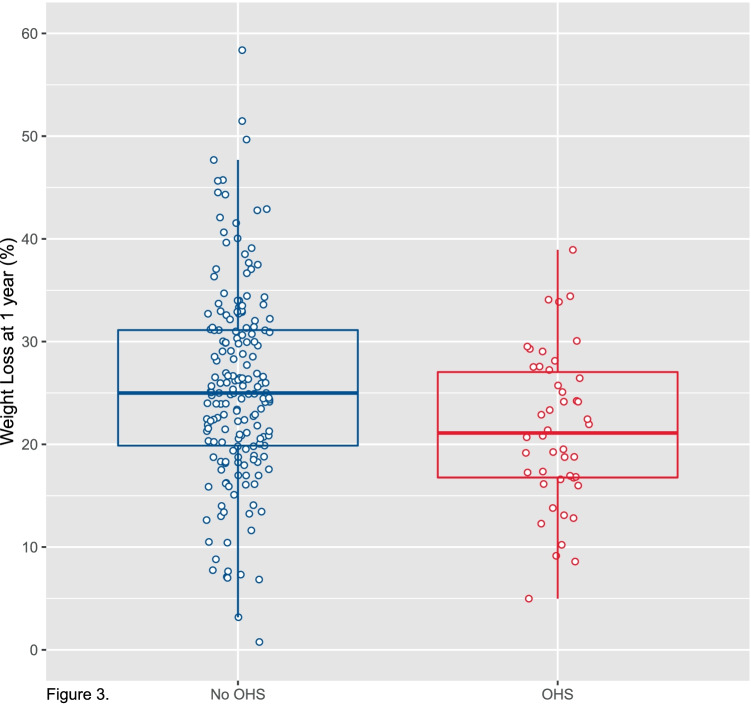
Weight loss in OHS vs non-OHS patients

As shown in Fig. 3, mean percent weight loss at 1 year was 21.5% (95% CI 19.25 to 23.7; *P* < 0.001) in suspected OHS patients compared with 25.7% (95% CI 24.3 to 27.0; *P* < 0.001) in non-elevated bicarbonate patients; i.e., mean weight loss was 4.2% (95% CI 1.6 to 6.8) less in suspected OHS vs non-OHS patients, which was noted to be statistically significant (*P* = 0.002). Results were similar when adjusting for baseline covariates including age in a multivariable regression analysis (mean weight loss 3.3% less in suspected OHS vs non-OHS patients, *P* = 0.03). Furthermore, a sensitivity analysis of 43 elevated bicarbonate cases who were age-matched with controls in a 1:3 ratio (mean [SD] age 45 [9] vs 45 [9] years) confirmed the robustness of these results (mean weight loss 4% less in OHS vs non-OHS patients, *P* = 0.005).

**Fig. 4:**
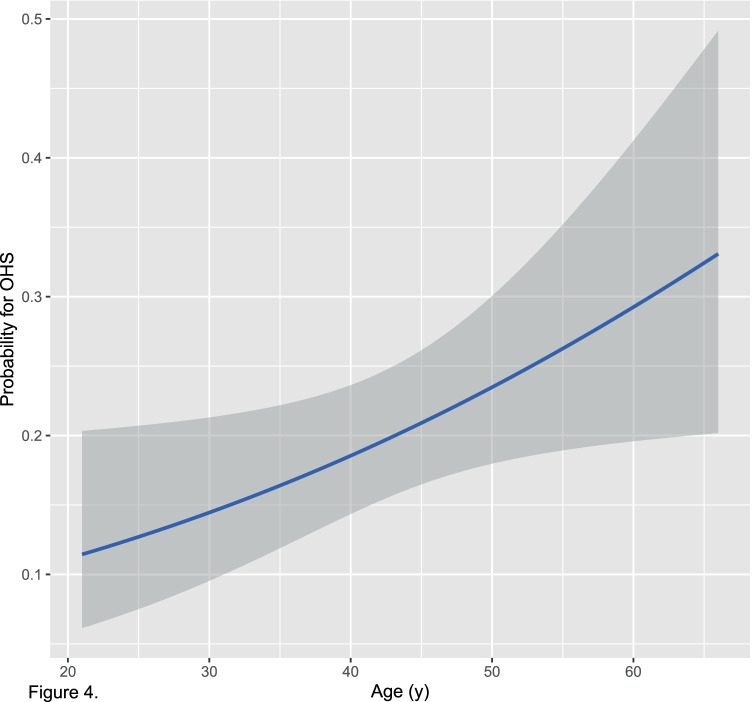
Predicted probability of OHS based on age (with 95% confidence interval)

In our cohort, age was the only significant predictor of elevated bicarbonate and suspected OHS (*OR*_per 10 year change_ = 1.44, 95% CI 1.02 to 2.07, *P* = 0.04); for every 10 years of age, the odds of suspected OHS increase by 44%. Figure 4 shows the predicted probability of suspected OHS for a given age.

## Discussion

The length of postoperative hospital stay in our group of patients undergoing bariatric surgery did not vary significantly based on preoperative serum bicarbonate levels, adding insight into the role of this surrogate marker for potential OHS. Furthermore, we observed a small but significant influence of elevated bicarbonate level on weight loss at 1 year. As a result, our findings did not support the notion that obesity hypoventilation syndrome (suspected based on surrogate measurements) led to deleterious perioperative consequences for those afflicted but did impact the longer-term weight loss results to some extent.

The mechanisms underlying potential poor outcomes in OHS are unclear. A number of studies have been conducted in this context. Some studies have suggested evidence of pulmonary hypertension, which could clearly contribute to worsened cardiovascular outcomes as well as exercise impairment [19, 23, 24]. While interventions such as noninvasive ventilation (and continuous positive airway pressure (CPAP)) have been shown to reduce hypoventilation and prevent morbidity from long-term hospitalizations, underdiagnosis remains a large barrier to providing this treatment [25]. Our study may be especially notable as the ATS Clinical Practice Guideline on OHS highlights bariatric surgery as one of the most viable options for the degree of sustained weight loss (20–35% total weight loss) that is needed to improve hypoventilation [6, 7]. In our study, patients with suspected OHS based on elevated serum bicarbonate exhibited a statistically significant smaller percent total body weight loss. Despite this finding, these patients were still able to obtain on average 21.4% total body weight loss, which is still within the clinically beneficial range.

Our study had strengths as well as novelty including a large sample size, careful outcome assessments, and study of a clinically important and underappreciated topic [26]. Nonetheless, we acknowledge several limitations. First, we did not directly measure arterial blood gases in our participants. Our goal was to perform a real-world study, and ABGs are rarely assessed in the preoperative evaluation in many centers. The reliance on serum bicarbonate for our study is imperfect but consistent with typical clinical practice. By excluding people receiving glucocorticoids and diuretics, we likely reduced the number of false positives which may have otherwise occurred. Second, we may have lacked statistical power to identify causes of postoperative complications since our event rates were quite low. We nevertheless believe that bariatric surgery can be safely performed in experienced academic medical centers and that our findings are consistent with those of other groups [27, 28]. Third, we assessed postoperative weight loss at 1 year, but did not rigorously gather data on diet and exercise patterns during the postoperative period. Additional factors such as presence or absence of diabetes, use of obesogenic medications, or use of weight loss medications may all have influenced these outcomes. Individual variances may not have been accounted for, but in aggregate, our data suggest that weight loss is relatively consistent for the various groups. Furthermore, while we feel follow-up 1 year after surgery has important clinical implications, we acknowledge that obesity is a chronic, lifelong illness and more research is needed to determine variances in sustained weight loss at even longer-term follow-up points. Additionally, it may be interesting in future studies to assess serial changes in bicarbonate levels in various cohorts undergoing different weight loss medical therapies and procedures. We also acknowledge that while 15–20% total body weight loss is a guideline-recommended weight loss [7], we did not evaluate for individual resolution of obesity-associated comorbidities such as diabetes, hypoventilation, or sleep apnea in our cohort. Despite these limitations, we believe that our findings are important and hope that they encourage further research in this area.

## Conclusion

Bariatric surgery can be safely performed in experienced medical centers. Preoperative elevated serum bicarbonate does not appear to be a major risk factor for perioperative complications or poor postoperative outcomes. Patients with elevated bicarbonate and suspected OHS may experience less weight loss but are still generally able to achieve clinically important outcomes. Our results suggest patients with elevated serum bicarbonate and suspected OHS patients should be encouraged to pursue bariatric surgery if appropriate for them independent of preoperative serum bicarbonate levels. Further work is needed to identify high-risk patients and to optimize postoperative outcomes in people with obesity.
